# A case report of peroneal muscle atrophy type 2A2 with central nervous system involvement as initial presentation

**DOI:** 10.1186/s12887-023-04441-z

**Published:** 2024-01-05

**Authors:** Xin Xu, Fen Lu, Senjie Du, Li Zhang

**Affiliations:** Department of Rehabilitation, Children’s Hospital of Nanjing Medical University, Guangzhou Road, Nanjing, Jiangsu 211103 China

**Keywords:** Charcot-Marie-Tooth disease type 2A2, MFN2, Cerebral nervous system

## Abstract

**Background:**

Charcot-Marie-Tooth disease (CMT) is a group of single-gene hereditary diseases of peripheral nerve with high clinical variability and genetic heterogeneity. The typical clinical manifestations include progressive muscle weakness and muscle atrophy in the distal extremities, accompanied by disappearance of tendon reflexes and distal sensory disturbances. CMT2A2 (OMIM: 609260) is caused by the mutation of MFN2 (OMIM: 608507), is the most common type of axonal pattern. Although a small number of patients with X-linked CMT1 (CMT1X) present with central nervous system involvement, including reversible white matter lesions, it is rarely in CMT2A2.

**Case presentation:**

A 3-year and 5-month-old girl had experienced motor lag, muscle tension, and abnormal gait for over a year. A reexamination of cranial MRI revealed an anterior enlargement of the abnormal signal range in the lateral ventricles and bilateral frontal lobes. And the whole exon sequencing showed that this girl carried a heterozygous missense mutation c.314C > T of MNF2 gene, inherited from her mother.

**Conclusions:**

In this study, we retrospectively analyzed the clinical and molecular genetic findings of a child with Charcot-Marie-Tooth disease A2 with central nervous system involvement as the initial presentation, and explored its pathogenic mechanism.

## Medical records

A 3-year and 5-month-old girl presented to Children’s Hospital Affiliated to Nanjing Medical University in October 2019 because of “motor backwardness, muscle tension and abnormal gait for more than 1 year”. Since childhood, the child suffered from motor developmental delay and was still unstable in walking alone at the age of 2 years old, accompanied by increased muscle tension in the left limb and inflexibility in the left limb when walking alone. As a result, “cerebral palsy (left hemiplegia)” was locally diagnosed and rehabilitation therapy was intermittently performed. When she was 3 years and 2 months old, she experienced motor dysfunction, manifested as right limb movement disorder, and she could not stand or walk alone. When the girl was helped to walk, she had hip flexion, knee abduction, and sharp enough, as well as mild internal rotation and varus of the foot, with high foot arch. Her language and cognition were also backward, and she can only send the single-character sound of “Dad and Mom”, and complete simple instructions. The patient was G4P2, and underwent cesarean section at term due to “oligohydramnios”. Her birth weight was 3500g, Apgar score was unknown, and she denied any rescue history of asphyxia. She had been insured during the first trimester of mother’s pregnancy, but there was nothing special about the rest. She had two previous history of “febrile convulsions”. Her parents were healthy, non-consanguineous and had a 7-year-old sister with normal athletic and intellectual development, who denied any similar medical history in her family.

Physical examination on admission: The body weight was 15 kg, the length was 97cm, and the head circumference was 50cm. She was conscious, and her reaction was moderate. Her bilateral pupils were equidistant and identical, and her reflex to light existed. There was no abnormality in physical examination of heart, lung and abdomen, and no abnormality in spine. Muscle strength of both upper limbs was normal, muscle strength of left lower limb was Class IV, and muscle strength of right lower limb was Class V. Muscular tone of both upper limbs was normal, and muscular tone of both lower limbs was increased, with obvious left side, bilateral ankle dorsiflexion limited, and dorsiflexion angle of foot-10. Pain and warmth sensation in the limbs is normal. Physiological reflex: hyperreflexia of bilateral knee tendons; pathological reflex: ankle clonus and Babinski sign (+). Due to high muscle tension and insufficient muscle capacity in the lower limbs, the girl had hip flexion, knee inversion, and pointy foot posture when she was helped to walk. So she walked with poor coordination and falled easily.

**Fig. 1:**
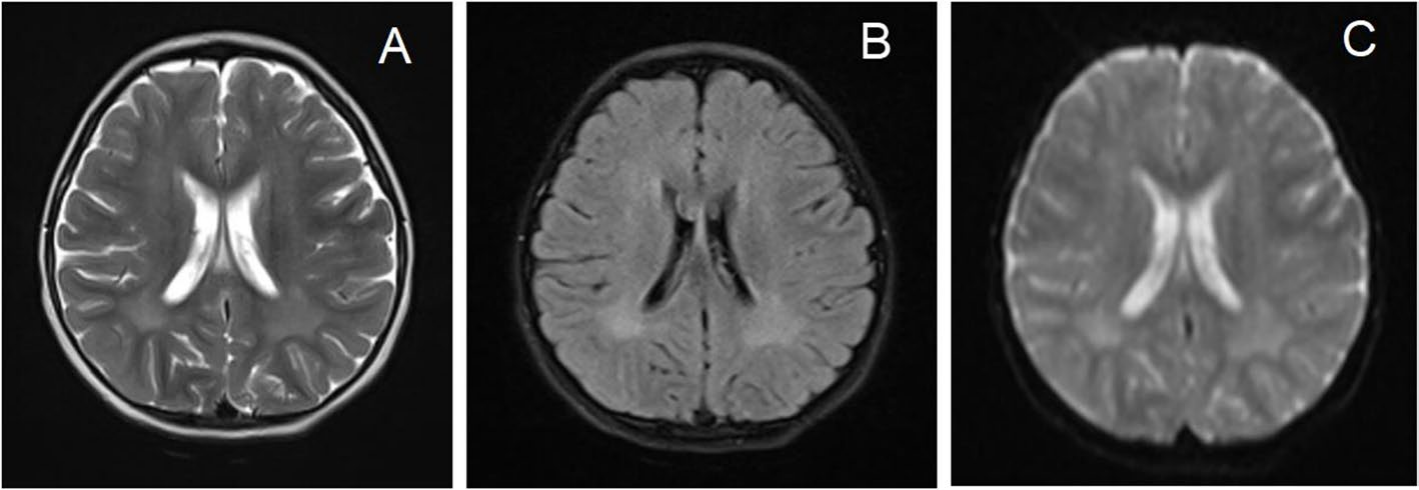
Cranial MRI results of children at the age of 3 years and 2 month. **A**. T2WI, **B**. Flair, **C**. DWI and T2WI images showed patchy high signal in bilateral lateral ventricles and bilateral frontal lobes. Flair was also high signal. No obvious diffusion limitation was observed on DWI

**Fig. 2:**
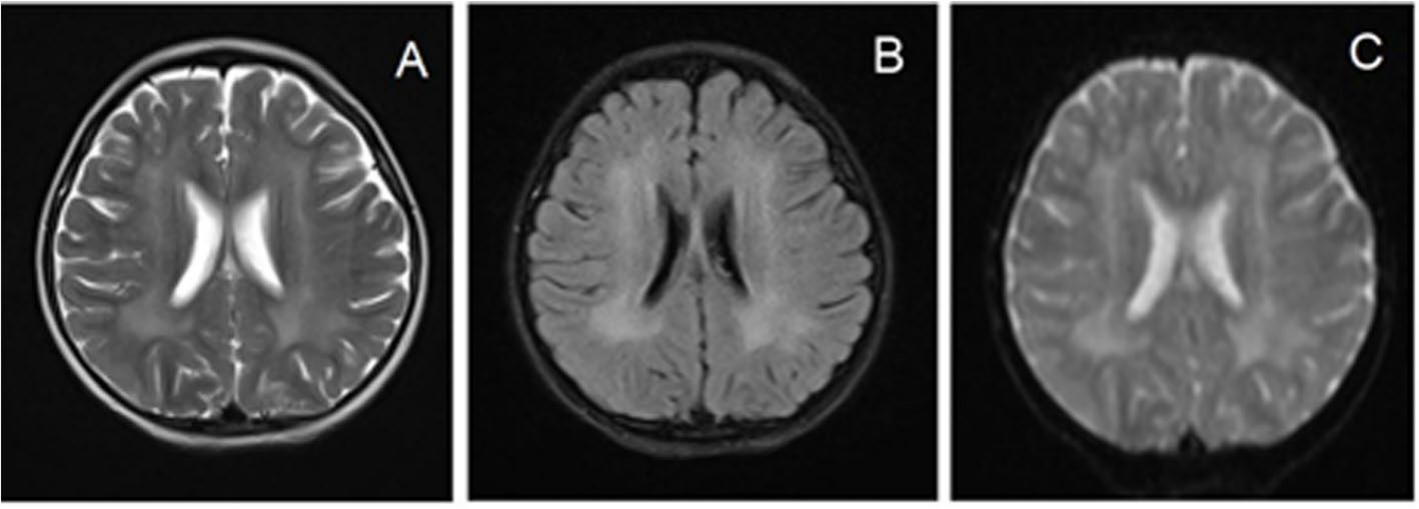
Cranial MRI results of children aged 3 years and 5 months. Images taken by **A**. T2WI, **B**. Flair, **C**. DWI, T2WI and Flair showed that the areas of high signal intensity in the lateral ventricles and patchy areas in the bilateral frontal lobes were larger than those before

Laboratory tests: routine blood test, liver and kidney function, electrolyte, and blood ammonia were normal. No abnormality is found in blood tandem mass spectrometry; Serum lactic acid: 2.3 mmol/L. No abnormality was found in the positive pelvic radiography and audio-visual evoked potentials. EEG showed a slowdown in background activity. Three months before admission, magnetic resonance imaging (MRI) of the head showed patchy long T2 signal shadows in the lateral ventricles and frontal lobes, high signal intensity in Flair, and no obvious diffusion limitation on DWI (Fig. 1). A reexamination of cranial MRI after admission revealed an anterior enlargement of the abnormal signal range in the lateral ventricles and bilateral frontal lobes (Fig. 2). No abnormality was found on MRI scan of spinal cord. The EMG examination showed that the amplitude of the bilateral tibial sensory nerve action potential (SNAP) was low, and all the observation indexes of the motor and sensory nerves in the remaining subjects were unremarkable. Tests with the Gesell Development Diagnostic Scale for children aged 0-6 years old showed that great exercise was equivalent to 10 months, fine movement was equivalent to 15 months, language was equivalent to 12 months, and adaptability and social behavior were equivalent to 21 months.

**Fig. 3:**
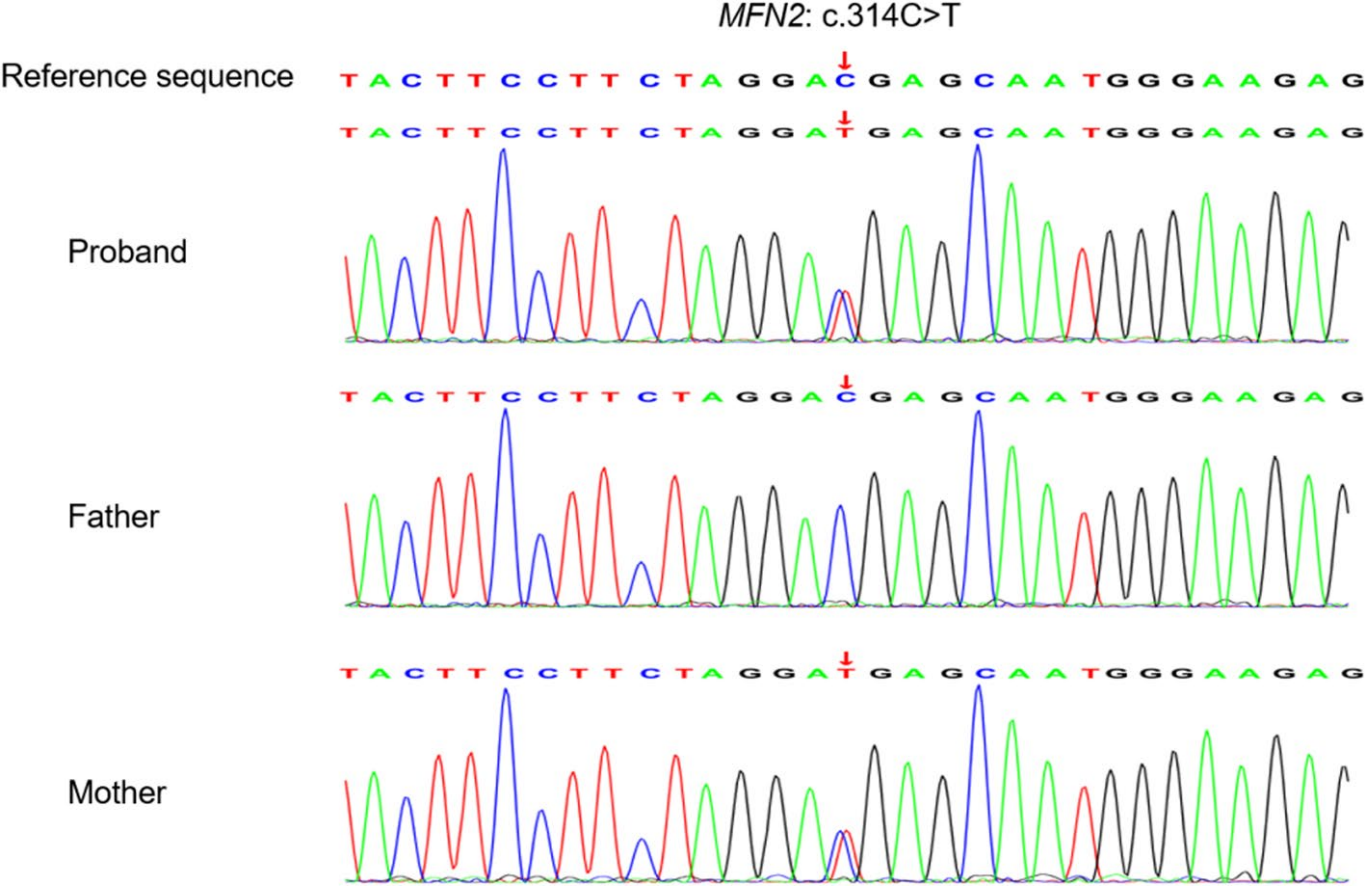
Sanger sequencing chromatograms of *MFN2* variant of the child and her parents and sister. Red arrows indicate the mutation site

Gene detection: the child had motor regression, the white matter lesion progressed, and genetic metabolic diseases could not be ruled out. The incidence rate of CMT is about 1/2500 [1]. To determine the cause, 4ml of peripheral blood from the child and 2ml of peripheral blood from their parents were collected after informed consent was signed by their parents for mitochondrial ring gene and whole exon group sequencing. The results showed that no pathogenic variation associated with the disease phenotype was found by sequencing the mitochondrial loop gene. However, whole exon group sequencing showed that the *MFN2* ($NM_014874.4$) of the child had the $c.314C>T$ heterozygous missense mutation, leading to the protein translation from threonine at position 105 to methionine (*p*.*T*105*M*). In Sanger validation analysis, her mother carried the mutation, and her father and sister had no abnormalities at the locus, and the mutation originated from the mother (Fig. 3). The mutation was analyzed according to the guidelines of the American College of Medical Genetics and Genomics (ACMG) [2]. The mutation of *MFN2* $c.314C>T(p.T105M)$ has been reported in patients with CMT2A2 [3, 4] (PS1). In vitro functional experiments have confirmed that this mutation can affect protein function [5, 6] (PS3); Minimal allele frequency $(MAF) < 0.0005$, belonging to low-frequency variation ($PM2_Supporting$); Multiple protein function prediction softwares (including SIFT, Polyphen2, Mutation Taster, and PROVEAN) predicted that the mutation would affect the gene product (PP3). The $c.314C>T(p.T105M)$ variant of the *MFN2* was defined as the pathogenic variant ($PS1+PS3+PM2_Supporting+PP3$) according to the ACMG guidelines.

**Fig. 4:**
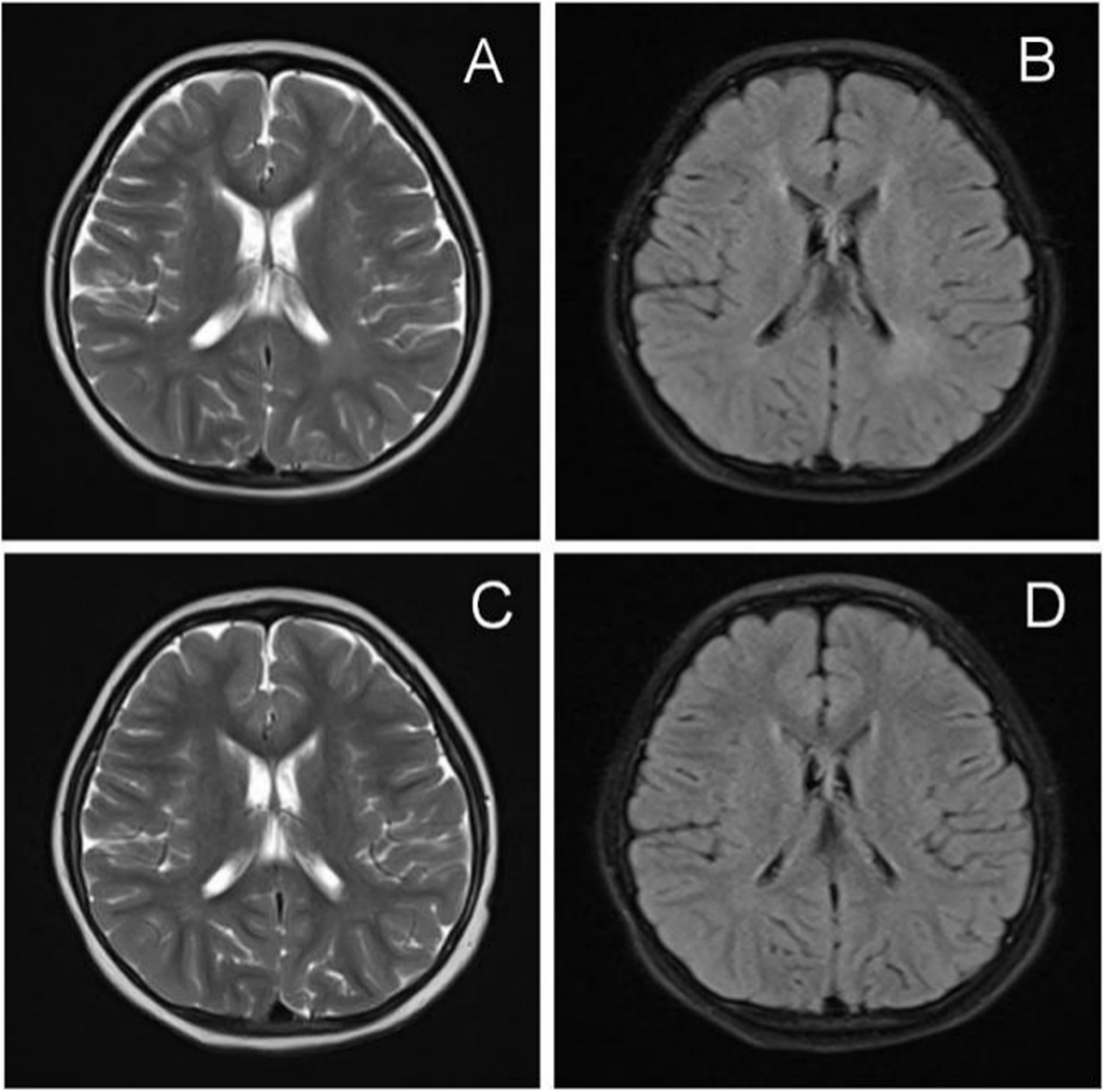
MRI results of 4-and 5-year-old heads of children. Images taken by **A**. T2WI, **B**. Flair, **C**. T2WI, **D**. Flair, T2WI and Flair showed that the areas of high signal intensity in bilateral lateral ventricles and patchy areas in bilateral frontal lobes were smaller than those at the time of admission

Clinical diagnosis and follow-up visit: The child was given comprehensive rehabilitation treatments such as exercise, occupational therapy and language training after admission, and then rehabilitation training was performed in our department according to the course of treatment. Another cranial MRI examination of the 4-year-old (Fig. 4A and B) and 5-year-old (Fig. 4C and d) children showed that the signal shadow scope of ventricular septal defect was smaller than that at the time of admission (Fig. 4). When the child was 4 years and 5 months old, the EMG was re-examined, and it was found that the amplitude of the compound muscle action potential (CMAP) of the left common peroneal nerve was low, the H reflex waveforms of the bilateral tibial nerves were poor, and the incubation period was slightly prolonged. The amplitude of the SNAP of the sensed sensory nerve in both lower limbs was low or decreased, suggesting the myoelectric changes of mild multiple peripheral neurogenic damage (mainly involving the sensory and motor axons of both lower limbs). Based on the results of EMG and genetic tests, the patient was finally diagnosed as CMT2A2 with early onset. Follow-up so far shows that the patient is 5 years and 7 months old, and her great motor ability has improved. She can walk alone for tens of meters with orthopedic shoes, and no significant atrophy of the distal muscles of her lower limbs has been observed.

**Table 1:** Clinical manifestations and MFN2 genotype of CMT2A2 patients with central nervous system

Case	Gender	Onset age	Visiting age	Phenotypic characteristics	Genotype	Source of variation	Cranial MRI findings	Other symptoms	Document
1	woman	3 years old	6 years old	Early hairstyle, severe	c.1090C>T, p.R364W	De novo	Right frontal lobe combined with periventricular white matter T2 high signal	lateral curvature	[11]
2	woman	23 years old	45 years old	Late hairstyle, mild	c.839G>A, p.R280H	father	Fusion and subcortical multiple T2 and FLAIR hyperintensities in the left parietal lobe	Pain, dysarthria, tremor, migraine	[11]
3	woman	25 years old	52 years old	Late hairstyle, mild	c.839G>A, p.R280H	mother	Multiple subcortical T2 and FLAIR high signals	Pain, dysarthria, tremor, migraine	[11]
4	man	20 years old	38 years old	Late hairstyle, mild	c.839G>A, p.R280H	mother	Multiple subcortical white matter lesions	Sensorineural hearing loss, pain, tremor	[11]
5	woman	42 years old	49 years old	Late hairstyle, mild	c.1085C>T, p.T362M	mother	Left subcortical T2 and FLAIR high signals without abnormal enhancement	Temporary loss of sensation	[11]
6	woman	50 years old	64 years old	Late hairstyle, mild	c.494A>G, p.H165R	not in details	Multiple subcortical white matter lesions with high signal intensity on T2 and FLAIR	Sensorineural hearing loss, tremor	[11]
7	man	10 years old	35 years old	Late hairstyle, mild	c.494A>G, p.H165R	mother	Multiple subcortical white matter lesions with high signal intensity on T2 and FLAIR	Sensorineural hearing loss, tremors, hoarseness	[11]
8	man	16 years old	26 years old	Late hairstyle, mild	c.380G>A, p.G127D	De novo	Multiple non-specific subcortical hyperintense lesions	Plantar extensor response	[11]
9	woman	38 years old	48 years	Late hairstyle	c.1392+2T>C	not in details	Diffuse high T2 signal in upper brainstem and periaqueductal gray	Subacute encephalopathy	[12]
10	man	3 years old	18 years old	Early hairstyle, severe	c.310C>T, p.R104W	De novo	Supratentorial white matter, mainly located in the peripheries of ventricles with mild diffuse T2 and FLAIR hyperintensities	Optic atrophy, megacephaly	[13]
11	man	7 years old	14 years old	Early hairstyle, severe	IVS5-1G>C	father	Slight high T2 signal intensity in the parietal occipital white matter and symmetrical high T2 signal intensity in the lateral part of bilateral thalamus	Big head deformity	[13]
12	woman	<2 years old	3 years old May	Early hairstyle	c.314C>T, p.T105M	mother	Patchy high signals of T2 and FLAIR in the lateral ventricle collateral and bilateral frontal lobe	Positive pyramidal tract sign	This example

Note: Case 2, case 3, and case 4 were members of the same family, and case 6 and case 7 were members of the same family

## Discussion

CMT2A2 is caused by a genetic variation of *MFN2* chromosome 1p36.22 *MFN2* consists of 20 exons and encodes a 757-amino-acid-containing mitochondrial fusion-2 (mFN2). *MFN2* is a transmembrane GTPase protein on the mitochondrial outer membrane, and has a GTPase domain, a first coiled-coil domain (heptapeptide repeat sequence HR1), two C-terminal transmembrane domains, and a second coiled-coil domain (heptapeptide repeat sequence HR2). Mitochondrium is a highly dynamic two-membrane organelle with different morphologies under the influence of metabolic conditions, development stages and environmental stimulation. The highly dynamic morphological changes of mitochondria are regulated and balanced by the fusion and division of mitochondrial membrane. *MFN2* is involved in the regulation of mitochondrial fusion process, as well as damaged mitochondrial repair, efficient mitochondrial energy metabolism, regulation of mitochondrial-endoplasmic reticulum calcium coupling, mitochondrial axon transport, and mitochondrial autophagy [7]. *MFN2* is commonly expressed, especially in tissues with high density of mitochondria, such as skeletal muscle and heart. $Mfn2^{-/-}$ homozygous mice died in the second trimester of pregnancy due to placental defect, and $MFN2^{+/-}$ heterozygous mouse embryonic fibroblasts had low mitochondrial motility [8]. Therefore, variation in the *MFN2* can causes mitochondrial dysfunction by affecting mitochondrial fusion and other processes.

The typical clinical manifestations of CMT2A2 patients are chronic progressive distal limb muscle weakness and muscle atrophy, which mainly affect the distal muscles of the lower limbs first. More than 90% of them have a drooping gait caused by tibialis anterior’s palsy. Another clinical feature is sensory loss, which mainly affects vibration perception. Neurophysiological studies have shown a decrease in the amplitude of the compound motor and sensory nerve action potentials in patients. Neuropathology in patients with CMT2A2 revealed chronic axonal degeneration and fibrotic regeneration clusters, a decrease in the number of myelinated fibers, and mitochondrial degeneration. Chronic axonal degeneration mainly affects the distal neurons of the longest peripheral nerve, and the most frequently proposed mechanism is that *MFN2* dysfunction causes interrupted mitochondrial transport and abnormal energy distribution of neuronal axons [9, 10]. Some patients with CMT2A2 may present with vocal cord paralysis, optic atrophy, sensorineural hearing loss, scoliosis, postural hand tremor, and pain.

Up till now, only a few foreign articles have reported CMT2A2 patients with central nervous system involvement [11–13] (Table 1). Chung et al. [11] reported that eight patients from five CMT2A2 families had white matter involvement, and the cranial MRI showed high T2 and Flair signals in the semioval center, periventricular and subcortical white matter, respectively. Only 1 patient presented with early onset (< 10 years old), while the other 7 patients presented with late onset (> 10 years old) and mild conditions, which could be combined with sensorineural hearing loss, pain, dysarthria, tremor and migraine. Boaretto et al. [12] reported a case of CMT2A2 with onset in adulthood and the most severe central nervous system involvement. The patient presented with fatal subacute encephalopathy manifestations such as vomiting, nystagmus, chorea, confusion and autonomic nerve dysfunction in clinical. The cranial MRI showed diffuse T2 high signal in the upper brain stem and periaqueductal gray. Although the literature has reported more frequent abnormal cranial MRI findings in patients with late onset, there is no clear correlation between the severity of central nervous system involvement and the age or clinical severity of onset. At present, the mechanism of central nervous system involvement of CMT2A2 is still unclear, which is speculated to be related to mitochondrial energy metabolism disorder in the brain caused by *MFN2* mutation.

MRI of the head of the child in this case showed white matter lesions in both lateral ventricles and frontal lobe, involving the paraventricular pyramidal tract. Therefore, the initial manifestations of the disease in the child were increased muscle tension in the lower limbs, abnormal gait and positive pyramidal tract sign. The early clinical manifestations were similar to those of cerebral palsy, and there was no manifestation of peripheral nerve involvement. After one and a half years of imaging follow-up, the white matter lesions in children gradually became smaller, which also supports that the white matter lesions in children may be due to insufficient mitochondrial energy supply. Because the central nervous system is a tissue with high mitochondrial density and metabolic needs, when stress occurs, mitochondrial energy metabolism is disrupted, resulting in insufficient energy supply. Lesions of the central nervous system may be reversible when the energy supply is restored.

To date, more than 100 pathogenic mutations of MFN2 in patients with CMT2A2 have been reported. Missense variation was the most common variation type (> 50%), followed by nonsense and missing variation [14]. Most of the mutations are located in or close to the GTPase domain and coiled-coil domain of MFN2protein. The patients who carry this type of mutation mostly suffer from early onset in childhood and have severe clinical symptoms. However, a small number of patients have mutations located at the carboxy-terminal region, whose usually have a later onset age and a milder disease [15]. A large sample case study in China identified 14 MFN2 missense mutations in nine sporadic and six CMT2A2 families [15]. Eight of these were within or near the GTPase domain, and five were new mutations. In addition, the common clinical feature of CMT2A2 patients in China is that they have an early onset, but the phenotypic severity varies, most of which is moderate to severe. However, CMT2A2 has strong clinical variability, and even among family members with the same MFN2 variation, there are individual differences in disease manifestation and progression [3]. There was also no significant correlation between the presence and severity of central nervous involvement and the type of MFN2 variation. The most frequently mutated amino acid in MFN2 protein is arginine at position 94 (including R94W and R94Q), which is a highly conservative residue and a hot mutation region upstream of GTPase domain [14]. In China, case reports of CMT2A2 families with MFN2 R94W and R94Q mutations have also been reported [15–18].

In this patient, a heterozygous missense variant of the $MFN2 c.314C>T(p.T105M)$ was detected, which is a known pathogenic variant located in the GTPase domain. Currently, all the patients with genotype *MFN2* T105M reported in the literature exhibit no signs of central nervous system involvement. Functional studies by multiple research groups have confirmed that this mutation is detrimental to *MFN2* protein function. Overexpression of the T105M mutant MFN2 protein in mouse fibroblasts can further induce mitochondrial aggregation by inhibiting mitochondrial fusion [5]. Bannerman et al. [19] knocked the T105M transgene with the STOP sequence flanked by loxp into mouse Rosa26 site, and hybridized these mice with nestin-Cre (expressed in neuroectoderm) transgenic mice to achieve specific expression of neuroectoderm Mfn2 T105M. Hybrid mice showed abnormal gait and muscle fiber atrophy in the calf muscles, as well as reduced mitochondria in the axons, which resembles the neuromuscular pathology in patients with CMT2A2.

At present, there is no specific treatment available for CMT2A2. Rehabilitation training, particularly aerobic training, orthotic devices to prevent joint deformation, and other symptomatic treatments are typically administered. In the late stage, patients with severe joint deformation can be given surgical intervention. After rehabilitation treatment, our patient’s physical fitness improved significantly, which was considered to be related to the amelioration of her cerebral white matter lesions. Despite being a chronic progressive disease, regular EMG reexamination is recommended during the later stages. The maternal inheritance of the MFN2 variant was observed in this patient, whose mother displayed only high arch without any clinical signs of peripheral nerve involvement. This is in line with the incomplete dominance of MFN2 variation, as up to 25% of carriers of MFN2 variant carriers from some families demonstrate normal electrophysiological examinations and merely display subclinical symptoms [4]. Therefore, genetic counseling remains necessary for future pregnancies involving this case’s parents.

In summary, a small subset of patients with CMT2A2 can present with initial central nervous system involvement, along with insignificant early peripheral nerve damage. This presents a diagnostic challenge and can easily be misdiagnosed as cerebral palsy. Gene testing helps to confirm the diagnosis at an early stage. The present study also expands the clinical phenotypic spectrum of the MFN2 T105M genotype in patients with CMT2A2.

## Data Availability

The original contributions presented in the study are included in the article; further inquiries can be directed to the corresponding author(s).

## Abbreviations

CMT: Charcot-Marie-Tooth
CMT1: Demyelinating type
CMT2: Axonal type
CMT1X: X-linked CMT1
CMT2A2: Charcot-Marie-Tooth disease A2
MRI: Magnetic resonance imaging
SNAP: Sensory nerve action potential
ACMG: American College of Medical Genetics and Genomics
MAF: Minimal allele frequency
CMAP: Compound muscle action potential
